# Cost analysis and resource allocation in the management of benign thyroid nodules: a comparison of surgery and thermal ablation techniques

**DOI:** 10.1007/s40618-025-02597-2

**Published:** 2025-05-22

**Authors:** Enrico Papini, Michele Basile, Roberto Novizio, Agostino Paoletta, Agnese Persichetti, Irene Samperi, Alessandro Scoppola, Anna Crescenzi, Annamaria D’Amore, Maurilio Deandrea, Andrea Frasoldati, Roberto Garberoglio, Rinaldo Guglielmi, Giovanni Mauri, Celestino Pio Lombardi, Soraya Puglisi, Teresa Rago, Vincenzo Triggiani, Dominique Van Doorne, Domenico Salvatore, Rosella Saulle, Roberto Attanasio

**Affiliations:** 1 Endocrinology, Ospedale Regina Apostolorum, Albano Laziale, Italy; 2 High School of Economy and Management of Health Systems, Catholic University of Sacred Heart, Rome, Italy; 3 Unit of Endocrinology, Fondazione Policlinico Universitario A. Gemelli- IRCCS, Catholic University of the Sacred Heart, Rome, Italy; 4 Endocrinology, ULSS6, Euganea, Padova, Italy; 5 Ministry of Interior - Department of Firefighters, Public Rescue and Civil Defense, Rome, Italy; 6 Endocrinology, ASL Novara, Rome, Italy; 7 Endocrinology, Ospedale Santo Spirito, Rome, Italy; 8 Department of Radiological, Oncological and Pathological Sciences, Sapienza University of Rome, Rome, Italy; 9 Division of Endocrine and Metabolic Surgery, Department of Medical Abdominal Surgical and Endocrine- Metabolic Sciences, Fondazione Policlinico Universitario A. Gemelli IRCCS, Rome, Italy; 10 Endocrinology and Center for Thyroid Diseases, Ospedale Mauriziano “Umberto I”, Turin, Italy; 11 Endocrinology, Arcispedale S. Maria Nuova IRCCS, ASL Reggio Emilia, Italy; 12 Private practice, Turin, Italy; 13 Interventional Radiology, Diagnostic and Interventional Radiology, IRCCS Ospedale Galeazzi-Sant Ambrogio, Italy; 14 Department of Clinical and Biological Sciences 1 st Internal Medicine, AOU San Luigi di Orbassano University of Turin, Orbassano TO, Italy; 15 Endocrinology, Department of Clinical and Experimental Medicine, University of Pisa, Pisa PI, Italy; 16 Department of Medicine, Endocrinology and Metabolic Diseases, University “Aldo Moro”, Bari, Italy; 17 AME Supervisor for Relationship with Patients’ Associations, Rome, Italy; 18 Department of Public Health, University Federico II, Naples, Italy; 19 Department of Epidemiology, Regional Health Service - ASL, Regione Lazio, Roma 1, Italy; 20 AME Scientific Committee, Milan, Italy

**Keywords:** Benign thyroid nodules, Cost-effectiveness analysis, Thermal ablation, Hemithyroidectomy, Total thyroidectomy, Italian healthcare system

## Abstract

**Introduction:**

Benign thyroid nodules are common in adults, with incidental detection rates reaching 50–60% in women. While most nodules are asymptomatic and require no treatment, some grow and cause compressive symptoms.

**Objective:**

This study evaluates the economic impact of various treatment modalities for benign thyroid nodules within the Italian healthcare system, comparing total thyroidectomy, hemithyroidectomy, and minimally invasive thermal ablation. The analysis considers both direct healthcare costs and indirect social costs, such as productivity losses, from the perspective of the Italian National Health Service.

**Methods:**

A literature review and clinician survey were conducted to collect cost data across treatment phases: pre-hospitalization, procedure, and post-operative care. An Activity-Based Costing approach was used to estimate full treatment costs, including medical expenses, staff involvement, material usage, and indirect costs related to work absences.

**Results:**

The findings indicate that total thyroidectomy has the highest overall cost (€ 5,185.36), followed by hemithyroidectomy (€ 4,211.92), and thermal ablation (€ 1,560.06). The analysis also highlighted significant cost savings associated with thermal ablation when compared to surgical options, especially in terms of reduced hospital stay and lower indirect costs. Nevertheless, surgical procedures remain the mainstay treatment due to long-term efficacy and well-established clinical guidelines.

**Conclusion:**

Thermal ablation represents a cost-effective alternative to surgery for managing benign thyroid nodules. Treatment selection should be individualized, considering clinical factors, patient preferences, and long-term outcomes. These results underscore the importance of updating management guidelines to incorporate economic considerations, promoting optimized care and efficient resource allocation in the healthcare context.

**Supplementary Information:**

The online version contains supplementary material available at 10.1007/s40618-025-02597-2.

## Introduction

Thyroid nodules are clinically detectable in 5–7% of the adult population, with a higher prevalence among females. As a result of preventive medicine policies, improved access to healthcare resources, and widespread use of imaging modalities such as ultrasound (US), computed tomography (CT), magnetic resonance imaging (MRI), and positron emission tomography (PET), the incidental finding of thyroid nodules is occurring with increasing frequency, with a prevalence reaching 50–60% in adult females [1–3]. 

The majority of thyroid nodules are cytologically benign and non-hyperfunctioning and, when clinically asymptomatic, do not require any therapeutic intervention. However, a small but significant proportion of benign nodules progressively increases in size and can cause local compressive symptoms, variable according to the size and anatomical location [4]. 

The therapeutic interventions for thyroid benign nodular pathology represent a high healthcare cost and the consequences of these treatments can negatively impact patients’ quality of life (QoL).

Evidence from North American databases show a progressive and constant increase in the use of thyroid surgery over the years (+ 39% from 1996 to 2006), with a significant rise in the proportion of total thyroidectomies performed for benign pathology (from 17.6% in the period 1993–1997 to 39.4% in the period 2003–2007), at the expense of more conservative interventions. European data follow the same trend: French registries document that nearly 60% of thyroid surgeries are performed for benign nodular pathology, while a German study shows that thyroidectomy for benign thyroid pathology is justified in only 30% of cases by the presence of compressive symptoms or hyperthyroidism [5–9]. 

The standard treatment for clinically symptomatic thyroid nodular disease is surgery. Thyroid surgeries are generally performed under general anesthesia and involve a cervical incision. Only exceptionally, a sternotomy is required due to mediastinal immersion of the goiter [10]. In recent decades, several non-surgical minimally invasive procedures have become available, all conducted under US guidance, aimed at reducing the size of benign nodules and resolving/ameliorating related symptoms. Notably, the long-term follow-up of these treatments generally covers up to 5 years, after which a partial regrowth of the nodule may occur in a minority of cases (10–15%), necessitating further therapeutic intervention. Minimally invasive techniques include chemical ablation with ethanol, also known as percutaneous ethanol injection (PEI), and thermo-ablation using laser (LTA), radiofrequency (RFA), microwave (MWA), and high-intensity focused ultrasound (HIFU) [11–13]. 

While hemithyroidectomy is a standardized therapeutic action alternative to total thyroidectomy, minimally invasive therapies with thermal ablation were more recently introduced, are less standardized, and require specific training for operators to be widely proposed nationwide. Overall, these techniques, performed in a day-hospital setting, offer an effective therapeutic outpatient option to reduce nodule volume and local symptoms without requiring general anesthesia. The risk of major permanent complications or loss of thyroid function is very low in specialized centers [14–16]. 

Due to the high prevalence of benign nodular pathology, it is necessary to optimize management algorithms based on the risk-benefit and cost-benefit ratios and on the level of accessibility to the different procedures. The patient must share the therapeutic choice with his/her doctor after being properly informed about the risk/benefit ratio and the impact of each option on his/her QoL.

## Objective

The primary objective of this study was to assess the cost implications associated with the different treatment options available for benign, symptomatic, non-hyperfunctioning thyroid nodules, aiming to complement the evidence on their clinical effectiveness. Total thyroidectomy, hemithyroidectomy and thermal ablation were considered as the alternative options.

The analysis was conducted from the perspective of the Italian National Health Service (NHS), integrated by a partial analysis of the social impact due to the productivity loss resulting from the absence of patients and their carers from work. A further objective of the analysis was to assess the cost-utility profile of these therapeutic alternatives to inform the health system policy making.

### Methods

#### Literature review

The analysis included a first phase aimed at gathering the available scientific economic evidence through a literature review considering resources involved in making recommendations.

A panel was established with the primary goal of developing Italian guidelines (GL) for the management of benign thyroid nodules that cause local symptoms. This multiprofessional and multidisciplinary panel was composed of highly experienced clinical professionals and played a pivotal role in shaping the GL. It included 12 endocrinologists, 4 endocrine surgeons, 3 radiologists, 1 pathologist, 1 health manager, 1 otolaryngologist, 1 nuclear medicine specialist, 1 general practitioner, and 1 psychologist [17]. 

The panel validated the PICO (Patient, Intervention, Comparison, Outcome) model for various research activities, including literature reviews, meta-analyses, and economic analyses. A systematic search was conducted on the following scientific databases: MEDLINE, Embase, CINAHL, and Web of Science (since inception to June 2020) to identify full economic evaluations (i.e., cost-effectiveness/cost-utility/cost-benefit analyses) of the interventions for benign, symptomatic, normally functioning thyroid nodules. The search strategy for MEDLINE is shown in Supplementary Material 1 [17]. 

## Survey

To determine the parameters useful for the considered interventions (hemithyroidectomy with isthmectomy, total thyroidectomy or thermal ablation), a questionnaire was prepared to collect information on specific drivers, including: the total duration of the intervention; the types of drugs used for the interventions, as well as their average dosage; the number and types of involved professionals, as well as the time amount employed by each of them to deliver the different interventions; the different materials used during the treatment administration (e.g., syringes, needle-electrodes, optical fibers) as well as the respective quantities; the percentage of patients assisted, according to the clinical experience of panel members, by a family member/caregiver, to determine productivity losses (indirect costs).

The questionnaire was filled in by the panel members. The economic analysis considered the average values of each parameter with the aim of generalizing the obtained results and making them compatible with the various contexts in which the NHS operates.

The analysis also considered the community perspective, by evaluating the support provided by caregivers/family members for the delivery of the interventions.

## Activity based costing analysis

The economic analysis was conducted using the methods of Activity-Based Costing [18]. This tool is useful for determining resource absorption and the subsequent evaluation of the full cost of the intervention being analyzed and involves three phases:

1. Identification of resources: this phase identifies the resources needed for the delivery of the therapies under consideration and distinguishes roles and timing in each phase, as well as the segments into which the process can be divided. This allows the cost related to each operation performed or unit of material used to be associated and the full cost of these sub-activities to be calculated. In this analysis, this phase was carried out by defining and administering an ad-hoc questionnaire.
2. Cost measurement: once the resources needed to provide the considered treatments were identified, their costs were quantified, referring to sources such as national price lists of drugs, disposable materials and salaries of the different professionals, as well as scientific literature.
3. Value definition: different cost drivers were assigned monetary values, allowing to determine the full value of each action performed and of the overall process.

The economic analysis considers four macro-categories of resource absorption related to the delivery of the therapies under analysis:

- Direct costs charged to the NHS for the delivery of considered treatments based on the type and dosage of the active employed drugs.
- Direct costs charged to NHS for the delivery of the considered treatments based on the numbers and types of disposable materials.
- Direct costs charged to the NHS for the delivery of the considered treatments based on the numbers and types of involved professionals.
- Indirect costs related to the productivity losses of the caregiver.

The results are therefore expressed as the full cost per patient treated of hemithyroidectomy with isthmectomy, total thyroidectomy, and thermal ablation.

## Results

### Cost drivers

Detailed information on preoperative procedures, drug usage, personnel involvement, and material costs is provided in Supplementary Tables 1–5.

## Pre-hospitalization procedures and structural costs

The questionnaire enabled the identification of the typical pre-hospitalization pathway for patients undergoing hemithyroidectomy with isthmectomy, thermal ablation, or total thyroidectomy. This included specialist consultations, diagnostic tests, and evaluations required before treatment, as well as follow-up visits and structural resources such as hospital stay and use of operating rooms. Detailed cost breakdowns of these components are provided in Supplementary Table 1 [19]. 

## Pharmacological therapy

The survey also collected data on the pharmacological agents administered during each procedure. This included the categories of drugs, dosages, and average costs per treatment. An overview of the drug-related expenses associated with each intervention is available in Supplementary Table 2 [20]. 

### Healthcare professionals and use of operating room

Information on the healthcare professionals involved in each procedure was gathered, including the number and type of staff (e.g., physicians, nurses, healthcare assistants, speech therapists), as well as the average time of involvement. The cost associated with the use of the operating room was also considered. These data are summarized in Supplementary Table 3.

### Consumable materials and medical devices

The analysis included the assessment of consumables and medical devices required for each intervention. This encompasses the quantity and cost of disposable materials, such as needles, surgical instruments, and ablation devices, specific to each treatment option. Full details are reported in Supplementary Table 4.

### Indirect costs and productivity loss

Indirect costs were estimated by evaluating productivity losses incurred by both patients and caregivers during the perioperative period. For the purpose of the analysis, it was assumed that all caregivers were employed full-time. The distribution of employment categories—managers, mid-level managers, employees, and workers/apprentices—was based on national data (Job Pricing Report, 2019) [21]. Productivity loss was calculated assuming five hours of work lost per day of therapy delivery, with a standard 40-hour work week across all employment categories. Specific assumptions and cost weightings used in the calculation are detailed in Supplementary Table 5.

### Total thyroidectomy

By weighting the consumption of the drivers considered in the analysis with respect to their unit cost, the analysis identified the total expenditure of the cost categories investigated relative to the implementation of total thyroidectomy.

Specifically, Table 1 reports the resource absorption in relation to the pre-hospitalization phase. This phase is associated with an average cost of € 281.80.

**Table 1:** Cost of pre-hospitalization procedures– total thyroidectomy

Procedure	Mean number of procedures performed in each patients	Weighted cost per procedure (€)
Specialist consultation	1.09	22.54
CBC	1.00	3.17
PT	1.00	2.85
PTT	1.00	2.85
Fibrinogen	1.00	2.67
ECG	1.00	11.62
Cardiology consultation	1.00	20.66
Otolaryngology consultation	0.90	18.59
Laryngoscopy	0.90	24.40
Neck ultrasound	1.00	28.41
Anesthesiology consultation	1.00	20.66
Thyroid fine-needle aspiration	0.90	77.49
Thyroid cytology exam	0.90	30.40
Chest X-ray	1.00	15.49
Total		281.80

Table 2 shows the average use per active ingredient and the cost per macro-category of drugs. The total pharmaceutical expenditure for each total thyroidectomy intervention is € 11.49.

**Table 2:** Cost of phamaceuticals– total thyroidectomy

Drug category	Drug	Dose per procedure (mg)	Cost per procedure (€)	Average category cost (€)
Anesthetics	Midazolam	0.75	0.10	1.54
	Propofol	175.00	2.58	
	Rocuronium	52.50	3.47	
Opioids	Fentanyl	0.21	0.62	6,30
	Remifentanil	3.94	11.97	
Antibiotics	Ceftriaxone	1,000.00	4.50	2.70
	Cefazolin	556.25	0.90	
Corticosteroids	Cortisone	2.00	0.04	0.41
	Betamethasone	4.00	0.77	
Other drugs	Paracetamol	1,000.00	0.37	
	Sodium chloride	1,000.00	0.18	
Total cost				11.49

Table 3 shows the results of the survey on the use of materials (devices, disposables, etc.) necessary for performing a total thyroidectomy. The average cost for delivering this intervention, in terms of the required instruments, was € 3,489.42, including the use of the operating room (€ 1,685.28) and hospital stay, estimated at 2.40 days, for a total cost of € 1,617.60.

**Table 3:** Cost of materials/devices used during total thyroidectomy

Material/Device	Mean number of items	Weighted cost per item (€)
Big clamp	2.33	58.41
Electrosurgical knife	1.00	14.34
Scalpel	2.00	30.77
Surgical strips	10.00	1.81
Suture thread	2.88	17.25
Gauze	9.38	0.93
Surgical drape	2.50	5.39
Dressing 8 × 15	1.00	0.54
Surgical swab	2.83	6.23
Sterile material pack	1.00	24.52
Vicryl suture	1.50	9.00
Monocryl suture	2.00	14.50
Jackson-Pratt drain	0.93	2.85
Hemovac drain	2.00	13.00
Operating room	1.00	1,685.28
Hospital stay (days)	2.40	1,617.60
Total		3,489.42

Table 4 shows the results of the questionnaire regarding the involvement of healthcare professionals during the performance of a total thyroidectomy. Specifically, the intervention involves, on average, two surgeons, 1.3 nurses, one anesthetist, 0.8 assistants (allied health professionals), and one surgical technician, engaged for an average time of 82.50 min. The determination of these parameters, weighted by the respective unit costs, allowed to calculate an average cost of delivering the intervention in terms of professional involvement of € 232.47.

**Table 4:** Cost of healthcare professionals involved during surgery and facility costs– Total thyroidectomy

Healthcare professional	Mean number of professionals	Weighted cost per professional (€)
Physician	2.00	95.58
Nurse	1.30	28.75
Anesthetist	1.00	47.79
Healthcare assistant	0.80	38.23
Surgical instrument technician	1.00	22.11
Total		232.47

The analysis investigated the follow-up phase of the patient who underwent total thyroidectomy, distinguishing between different post-operative courses:

- standard.
- with acute complications.
- with chronic complications.

According to the survey, the frequency of standard follow-up was 93% and 3.5% for patients with acute and chronic complications, respectively. The analysis revealed that the average annual cost associated with the standard post-operative course was € 143.04, and € 258.97 and € 297.22 for the post-operative course of patients with acute and chronic complications, respectively. (Table 5).

**Table 5:** Cost of post-operative follow-up– Total thyroidectomy

Follow-up category	Procedure	Mean number of procedures	Weighted cost per procedure (€)
Standard post-operative course	Specialist consultation	1.25	25.83
	CBC	0.33	1.06
	Serum Calcium	1.00	1.13
	TSH	1.00	5.46
	FT4	1.00	6.36
	Histological exam	1.00	27.17
	Neck ultrasound	1.00	28.41
	Levothyroxine therapy	N/A	47.63
Acute complication course	Endocrinology consultation	1.88	38.74
	Surgical consultation	1.50	30.99
	Serum Calcium	1.00	1.13
	Calcium infusion	0.50	1.78
	CBC	1.17	3.70
	Neck ultrasound	1.38	39.06
	Histological examination	0.78	21.13
	Video-laryngoscopy	1.00	27.11
	FT4	1.00	6.36
	Levothyroxine therapy	N/A	47.63
	Cost of reintervention	0.01	37.46
Chronic complication course	Endocrinology consultation	2.00	41.32
	Surgical consultation	1.25	25.83
	Speech therapy	7.50	110.42
	FT4	1.00	6.36
	CBC	1.00	3.17
	Histological examination	1.00	27.17
	Video-laryngoscopy	1.00	27.11
	Calcium/vitamin D	1.00	3.57
	Levothyroxine therapy	N/A	47.63
Standard post-operative course	Total		143.04
Acute complication course	Total		258.97
Chronic complication course	Total		297.22

The analysis of the costs associated with the delivery of a total thyroidectomy also included the productivity losses of the patient and his/her caregiver associated with the intervention. Specifically, the analysis revealed that on average, 5% of patients undergoing the intervention are assisted by a caregiver and that the average number of days of convalescence following the procedure is 11.22 days. Considering the total duration of the operation (13.62 days, of which 2.40 of hospitalization and 11.22 of convalescence) and a weighted hourly productivity loss of € 14.05, total thyroidectomy is associated with a weighted total productivity loss of € 1,004.68, of which € 47.84 for the caregiver and € 956.84 for the patient him/herself, assuming a number of working hours lost per day of 5.

Table 6 shows the various cost items associated with performing a total thyroidectomy. The highest cost is associated with the surgery (€ 3,746.39). The total cost of total thyroidectomy is therefore € 5,185.36.

**Table 6:** Comparative summary of total costs per procedure

Total cost of procedure– Total thyroidectomy	
Category	Weighted cost (€)
Pre-hospitalization	281.80
Pharmacological therapy	11.49
Materials	199.54
Healthcare professionals	232.47
Operating room	1,685.28
Hospital stay	1,617.60
Subtotal for surgery	3,746.39
Standard post-operative course	133.03
Course with acute complications	9.06
Course with chronic complications	10.40
Subtotal for follow-up	152.50
Productivity loss	1,004.68
Total intervention cost	5,185.36

Total cost of procedure– Thermal ablation	
Category	Cost (€)
Pre-hospitalization	369.25
Pharmacological therapy	1.94
Materials	661.98
Healthcare professionals	40.64
Hospital stay	193.78
Subtotal for the procedure	898.34
Standard post-operative course	91.11
Subtotal for follow-up	108.70
Productivity loss	183.77
Total procedure cost	1,560.06

Total cost of procedure– Hemithyroidectomy	
Category	Weighted ost (€)
Pre-hospitalization	281.80
Pharmacological therapy	12.08
Materials	149.97
Health professionals	184.65
Operating room	1,356.98
Hospital stay	1,348.00
Subtotal for surgery	3,051.77
Standard post-operative course	105.34
With acute complications	7.98
With chronic complications	9.07
Subtotal for follow-up	49.79
Productivity loss	755.97
Total intervention cost	4,211.92

Subtotal for follow-up reflects a weighted average of standard follow-up and follow-up with acute and chronic complications: 93% standard, 3.5% acute complications, 3.5% chronic complications.

### Thermal ablation

By weighting the consumption of the drivers considered in the analysis with respect to their unit cost, the analysis identified the total expenditure of the cost categories investigated relative to the implementation of thermal ablation. Specifically, Table 7 shows the resource absorption related to the pre-hospitalization phase. This phase is associated with an average cost of €321.09.

**Table 7:** Cost of pre-hospitalization procedures– thermal ablation

Procedure	Mean number of procedures performed in each patient	Cost per procedure (€)
Specialist consultation	1.00	20.66
Otolaryngology consultation	0.70	14.46
Laryngoscopy	0.11	3.01
Complete blood count (CBC)	2.10	6.65
Electrocardiogram (ECG)	0.70	8.13
Neck ultrasound	1.00	28.41
Thyroid fine-needle aspiration	2.00	172.20
Thyroid cytology exam	2.00	67.56
Total		321.09

Regarding the consumption of resources in terms of use of pharmacological alternatives, Table 8 shows the average use per active ingredient and the cost per macro-category of drugs. As can be observed, the analysis reveals that the class associated with the highest cost is anaesthetics, with a cost of €1.34 per procedure. The total pharmaceutical expenditure for each thermal ablation is € 1.69.

**Table 8:** Cost of pharmacological therapy used during thermal ablation

Drug category	Drug	Dose per procedure (mg)	Cost per procedure (€)
Anesthetics	Lidocaine	67.50	0.11
	Midazolam	4.00	0.53
	Ropivacaine hydrochloride monohydrate	7.50	3.38
Corticosteroids	Cortisone	9.33	0.17
Antibiotics	Ceftriaxone	0.33	0.00
Other drugs	Sodium chloride	400.00	0.07
	Paracetamol	500.25	0.19
Average anesthetics Cost			1.34
Average corticosteroids cost			0.17
Total cost			1.69

Table 9 shows the results of the survey on the use of materials (devices, disposable materials, etc.) necessary for performing a thermal ablation. The average cost for performing this procedure, from the point of view of the required instruments, was €744.15, including hospital stay estimated at 0.25 days, for a total cost of € 168.50.

**Table 9:** Cost of materials/devices used during thermal ablation

Material/Device	Number of Items	Cost per Item (€)
Gauze	7.38	0.73
Surgical drape	2.00	4.32
Surgical swab	2.40	5.28
Sterile material pack	0.89	21.80
Laser fibers	2.00	330.00*
Radiofrequency needle electrode	1.00	620.00*
Ultrasound machine	1.00	56.00
Ice pack	2.00	12.42
Anesthesia needle	1.00	0.06
ECG monitoring electrodes	1.00	0.04
Hospital stay	0.25	168.50
Total		755.15

Table 10 shows the results of the questionnaire regarding the involvement of healthcare professionals during the performance of a thermoablation procedure. Specifically, the procedure involved an average of 1.3 surgeons, one nurse and 0.83 assistants (allied health professionals) for an average time of 23.50 min. The determination of these parameters, weighted by the respective unit costs, allowed the calculation of an average cost of the procedure in terms of professional involvement of € 35.34.

**Table 10:** Cost of healthcare professionals and facility involved during thermal ablation

Healthcare professional	Number of professionals	Cost per professional (€)
Physician	1.30	17.70
Nurse	1.00	6.30
Healthcare assistant	0.83	11.34
Total		35.34

Finally, the analysis investigated the follow-up phase of the patient undergoing a thermal ablation procedure. The analysis revealed that the average annual cost associated with follow-up was € 97.97 (Table 11).

**Table 11:** Cost of Post-operative Follow-up– Thermal ablation

Follow-up category	Procedure	Number of procedures	Cost per procedure (€)
Standard post-operative course	Specialist consultation	1.73	35.69
	Thyrotropin (TSH)	1.00	5.46
	Neck ultrasound	2.00	56.82
Post-operative course	Total		97.97

The cost analysis associated with performing a thermal ablation procedure also included the productivity losses of the patient and his/her caregiver. Specifically, the analysis revealed that on average, 10% of patients undergoing the procedure are assisted by a caregiver and that the average number of days of convalescence following the procedure is 1.82. Considering the entire period involved in the procedure (2.07 days, of which 0.25 of hospitalization and 1.82 of convalescence) and a weighted hourly productivity loss of € 14.05, thermal ablation is associated with a total weighted productivity loss of € 159.80, of which € 14.53 for the caregiver and € 145.27 for the patient, assuming a number of working hours lost per day of 5.

Table 6 shows the various cost items associated with performing a thermal ablation procedure, including a 15% of patients who need to undergo the thermal ablation procedure again. As can be observed, the highest cost is associated with the procedure (€ 898.34), while the post-operative follow-up has a less significant impact on the overall expenditure. The total cost of the thermal ablation is therefore € 1.560,06.

### Hemithyroidectomy

Finally, by weighting the consumption of the drivers considered in the analysis with respect to their unit cost, the analysis identified the total expenditure of the cost categories examined in relation to the performance of hemithyroidectomy with isthmectomy.

Specifically, Table 12 shows the resource absorption related to the pre-hospitalization phase. This phase is associated with an average cost of € 281.80.

**Table 12:** Cost of pre-hospitalization procedures– hemithyroidectomy

Procedure	Mean number of procedures performed in each patient	Cost per procedure (€)
Specialist consultation	1.09	22.54
Complete blood count (CBC)	1.00	3.17
Prothrombin time (PT)	1.00	2.85
Partial thromboplastin time (PTT)	1.00	2.85
Fibrinogen	1.00	2.67
Electrocardiogram (ECG)	1.00	11.62
Cardiology consultation	1.00	20.66
Otolaryngology consultation	0.90	18.59
Laryngoscopy	0.90	24.40
Neck ultrasound	1.00	28.41
Anesthesiology consultation	1.00	20.66
Thyroid fine-needle aspiration	0.90	77.49
Thyroid cytology exam	0.90	30.40
Chest X-ray	1.00	15.49
Total		281.80

Table 13 shows the average use per active ingredient and the cost per macro-category of drugs. The total pharmaceutical expenditure for each hemithyroidectomy with isthmectomy intervention is € 12.08.

**Table 13:** Cost of pharmacological therapy used during hemithyroidectomy

Drug category	Drug	Dose per procedure (mg)	Cost per procedure (€)	Average category cost (€)
Anesthetics	Midazolam	1.50	0.20	1.56
	Propofol	175.00	2.58	
	Rocuronium	52.50	3.47	
Opioids	Fentanyl	0.21	0.62	6.30
	Remifentanil	3.94	11.97	
Antibiotics	Cefazolin	2,000.00	3.24	3.24
Corticosteroids	Cortisone	4.00	0.07	0.42
	Betamethasone	4.00	0.77	
Other drugs	Paracetamol	1,000.00	0.37	
	Sodium chloride	1,000.00	0.18	
Total Cost			12.08	

Table 14 shows the results of the survey on the use of materials (devices, disposables, etc.) necessary for performing a hemithyroidectomy with isthmectomy. The average cost for delivering this intervention, from the perspective of the required instruments, amounted to € 2,854.95, including the use of the operating room (€ 1,356.98) and hospital stay, estimated at 2.00 days, for a total cost of € 1,348.00.

**Table 14:** Cost of materials/devices used during hemithyroidectomy

Material/Device	Mean number of Items	Cost per Item (€)
Big Clamp	3.00	75.10
Electrosurgical knife	1.00	14.34
Suture thread	3.00	18.00
Gauze	13.00	1.28
Surgical drape	3.25	7.01
Surgical swab	3.25	7.15
Sterile material pack	1.00	24.52
Jackson-Pratt drain	0.83	2.56
Operating room	1.00	1,356.98
Hospital stay	2.00	1,348.00
Total		2,854.95

Table 15 shows the results of the questionnaire regarding the involvement of healthcare professionals during the performance of a hemithyroidectomy with isthmectomy. Specifically, the intervention involves, on average, 2.14 surgeons, 1.14 nurses, one anesthetist, 0.67 assistants (healthcare workers), and one surgical technician, engaged for an average time of 66.43 min. The determination of these parameters, weighted by the respective unit costs, allowed to calculate an average cost of delivering the intervention in terms of professional involvement of € 184.75.

**Table 15:** Cost of healthcare professionals and facility involved during hemithyroidectomy

Healthcare Professional	Mean number of Professionals	Cost per professional (€)
Physician	2.14	82.46
Nurse	1.14	20.35
Anesthetist	1.00	38.48
Healthcare assistant	0.67	25.65
Surgical instrument technician	1.00	17.81
Total		184.75

The analysis investigated the follow-up phase of the patient who underwent a hemithyroidectomy with isthmectomy, distinguishing between different post-operative courses:

- standard;
- with acute complications;
- with chronic complications.

According to the survey, the frequency of 93% was assigned to the standard follow-up and 3.5% each to the follow-up of patients with acute or chronic complications. The analysis revealed that the average annual cost associated with the standard post-operative course is equal to € 113.26, and € 227.91 and € 259.13, for acute and chronic complication follow-up, respectively (Table 16).

**Table 16:** Cost of Post-operative follow-up - Hemithyroidectomy

Follow-up category	Procedure	Mean number of procedures	Cost per procedure (€)
Standard post-operative course	Specialist consultation	1.50	30.99
	Complete blood count (CBC)	0.25	0.79
	Calcium test	2.00	2.26
	Thyrotropin (TSH)	2.00	10.92
	Free thyroxine (FT4)	2.00	12.73
	Histological exam	1.00	27.17
	Neck ultrasound	1.00	28.41
Acute complication course	Specialist consultation (endocrinology/otorhinolaryngology)	2.40	49.58
	Surgical consultation	1.80	37.19
	Calcium test	2.00	2.26
	Calcium infusion	0.50	1.78
	Complete blood count (CBC)	1.17	3.17
	Thyrotropin (TSH)	1.00	5.46
	Neck ultrasound	1.60	45.46
	Histological exam	1.00	27.17
	Video-laryngoscopy	1.00	27.11
	Cost of reoperation	0.01	30.75
Chronic complication course	Endocrinology consultation	2.80	57.85
	Surgical consultation	1.60	33.06
	Speech therapy	7.50	110.42
	Calcium test	1.00	1.13
	Thyrotropin (TSH)	2.00	10.92
	Histological exam	1.00	27.17
	Neck ultrasound	1.20	18.59
Standard post-operative course	Total		113.26
Acute complication course	Total		227.91
Chronic complication course	Total		259.13

The cost analysis associated with hemithyroidectomy with isthmectomy also included the loss of productivity of the patient and his/her caregiver. Specifically, the analysis revealed that, on average, 2.50% of patients undergoing the intervention are assisted by a caregiver and that the days of convalescence following the intervention are on average 8.50. Considering the entire period involved in performing the intervention (10.50 days, of which 2.00 of hospitalization and 8.50 of convalescence) and a weighted hourly productivity loss of € 14.05, the hemithyroidectomy with isthmectomy is associated with a weighted total productivity loss of € 755.97, of which € 18.44 for of the caregiver and € 737.53 for the patient, assuming a number of working hours lost per day of 5.

Table 6 shows the different cost items associated with performing a hemithyroidectomy with isthmectomy. As can be observed, the highest cost is associated with the surgery (€ 3,051.77), while the procedures related to post-operative follow-up have a less significant impact on the overall expenditure. The total cost of hemithyroidectomy with isthmectomy thus is € 4,211.92.

### Summary and comparison of treatment costs

Overall, the average total cost per patient was €5,185.36 for total thyroidectomy, €4,211.92 for hemithyroidectomy with isthmectomy, and €1,560.06 for thermal ablation. The most significant cost components for both surgical procedures were related to hospital stay, operating room use, and personnel, while thermal ablation was primarily characterized by device-related costs, with a substantially reduced need for hospitalization and faster recovery. In terms of indirect costs, productivity losses were also higher for total thyroidectomy (€1,004.68) and hemithyroidectomy (€755.97), compared to thermal ablation (€183.77). Combining direct and indirect costs, thermal ablation resulted in a cost reduction of approximately 70% compared to total thyroidectomy, and over 60% compared to hemithyroidectomy, without including additional long-term complications or recurrence rates. This comparative analysis highlights the significant economic advantage of thermal ablation, especially when performed in experienced centers and in appropriately selected patients.

## Discussion

The aim of this analysis was to determine, with the highest level of accuracy, the costs associated with the performance of surgical thyroidectomy, hemithyroidectomy, and thermal ablation of thyroid nodules. The results reveal that the average resource absorption per patient managed with total thyroidectomy, thermal ablation, and hemithyroidectomy with isthmectomy is € 5,185.36, € 1,560.06, and € 4,211.92, respectively. In addition, from the perspective of lost productivity by the patient and his/her caregiver, these interventions involve a consumption of resources equal to € 1,004.68, € 183.77, and € 755.97, respectively.

The data used to estimate resource absorption were obtained from a survey that meticulously investigated the drugs, materials, and health professionals involved in the care process, and the caregiver/family member contribution to the therapy. Among the costs considered, the intervention itself had the highest resource absorption for all the alternatives, representing 72.24%, 57.58%, and 72.45% of the total treatment costs for total thyroidectomy, thermal ablation, and hemithyroidectomy with isthmectomy, respectively.

A notable consideration in this analysis is the interpretation of personnel costs as “opportunity cost”, which reflects the resources, especially the time of health professionals, that must be allocated to these therapeutic strategies. From the perspective of NHS, healthcare personnel are paid regardless of service delivery. Therefore, the “real” cost, also from a societal perspective (including indirect costs), is the total reported cost minus personnel costs, which is € 4,952.90 for total thyroidectomy, € 1,519.42 for thermal ablation, and € 4,027.17 for hemithyroidectomy with isthmectomy.

These results, particularly the lower cost of thermal ablation, are in line with previous studies [16] that demonstrated cost savings in the management of symptomatic benign thyroid nodules following the introduction of thermal ablation as an alternative treatment in Italian hospitals. However, some limitations in the accuracy of cost calculations will require verification in clinical practice. For example, fluctuations in the prices of materials used in thermal ablation and surgery may influence costs. Additionally, the reported risk of recurrence for ablative procedures, that requires a further treatment, is approximately 5–25% [22–25] but could be higher in real-world settings, especially if such procedures are performed outside specialized centers.

This cost analysis assumes the same follow-up time for the three procedures, potentially underestimating the costs for ablative procedures. Clinical guidelines [11–13] suggest repeated ultrasound control for nodules treated with ablative procedures—at 1, 6, and 12 months after treatment and annually thereafter — more frequently if a dominant nodule in a multinodular goiter was treated. In contrast, for hemithyroidectomy, ultrasound of the intact residual lobe is generally performed cautiously at one year and subsequently as in patients with hypothyroidism. Furthermore, the cost of L-T4 therapy and other replacement therapies required in case of chronic surgical complications, together with the cost of monitoring and follow-up induced by these complications, should be verified over time. It is also likely that the real cost of personnel in surgical interventions is underestimated due to the exclusion of unproductive times between procedures, such as dressing and undressing, providing information on intervention modalities and possible complications, obtaining informed consent, cleaning the operating room, and monitoring the patient during weaning from anesthesia.

An important consideration when evaluating the cost-effectiveness of the compared treatments is the impact of the learning curve and the procedural volume of the centers. Operator experience and institutional procedural volume are key determinants of both clinical outcomes and cost-efficiency for thermal ablation as well as surgical interventions. In centers with higher case volumes and structured training programs, thermal ablation procedures tend to be more standardized, quicker, and associated with fewer complications, which translates into a more favorable economic profile [26, 27]. Similarly, the surgical management of thyroid nodules shows variability in complication rates, operating time, and resource utilization depending on the surgeon’s expertise and the institutional setting [28, 29]. Thus, the costs measured in our analysis—particularly for thermal ablation—may vary considerably in less experienced or low-volume centers, where longer procedural times, increased need for reintervention, or higher complication rates may affect the cost-benefit balance. Future cost analyses should account for this variable and these procedures could be mainly referred to centers with specific expertise to maximize both clinical and economic outcomes.

The adoption of the recommendations of this guideline [17] could lead to changes in the Essential Levels of Care within the NHS. In particular, a reduction in the annual number of surgical interventions in ordinary hospitalization for benign nodular pathology — currently the main cause of thyroidectomy — is expected in favor of minimally invasive treatments performed in day hospitals without the need for an operating room. This change should also reduce the occupancy rate of surgical beds and improve accessibility to specialized surgical departments for oncological interventions.

Finally, the reduction in the indirect costs related to the long-term replacement therapy for post-surgical hypothyroidism and to the management of the potential complications of surgery is expected.

### Conclusion and future perspectives

This study highlights the significant cost differences among the different treatment options for benign symptomatic thyroid nodules. Thermal ablation is a cost-effective alternative compared to hemithyroidectomy and total thyroidectomy. Surgical procedures remain the cornerstone in current clinical practice due to their long-term efficacy and technique standardization but the adoption of minimally invasive approaches could lead to considerable savings for the healthcare system and to improved patient quality of life. Future research should aim to validate these findings in real-world clinical settings, to monitor long-term outcomes of thermal ablation, and to assess the impact of repeated procedures and training-related costs. The development of updated national guidelines integrating both clinical and economic evidence is expected to ensure optimal resources allocation and personalized patient care.

## Electronic Supplementary Material

Below is the link to the electronic supplementary material.

Supplementary Material 1 (DOCX 72131 KB)
