# Supplementary material for: Cost analysis and resource allocation in the management of benign thyroid nodules: a comparison of surgery and thermal ablation techniques
Source: J Endocrinol Invest. 2025 May 22;48(8):1769–80. doi: 10.1007/s40618-025-02597-2 (PMC12313836; doi:10.1007/s40618-025-02597-2)
Supplement: Supplementary file 1 — Supplementary Material 1 (DOCX 72131 KB) [file 40618_2025_2597_MOESM1_ESM.docx]

**Supplementary Material 1**

Database: The Cochrane Library

Data della ricerca: Issue 6, 2020

Search Query:

MeSH descriptor: [Thyroid Nodule] explode all trees

MeSH descriptor: [Goiter, Nodular] this term only

(thyroi* NEAR (nod* or incidentalom* or goiter))

,ab,kw

((goiter* or goitre*) near (nodul* or multinodul* or multi nodul* or nontoxic or non toxic))

,ab,kw

Combined search: (#1 OR #2 OR #3 OR #4)

MeSH descriptor: [Thyroidectomy] explode all trees

thyroidectom*

,ab

surgery

,ab,kw

Combined search: (#6 OR #7 OR #8)

Final search: (#5 AND #9)

Database: Ovid MEDLINE(R)

Data della ricerca: 1946 to June 08, 2020

Search Query:

exp Thyroid Nodule/

exp Goiter, Nodular/

(thyroi* adj6 (nod* or incidentalom* or goiter)).tw,ot.

((goiter* or goitre*) adj6 (nodul* or multinodul* or multi nodul* or nontoxic or non toxic)).tw.

((thyroid adj3 (neoplasm or tumor)) and benign).tw.

Thyroidectomy/

thyroidectom*.tw.

Combined search: (1 or 2 or 3 or 4 or 5)

Combined search: (6 or 7)

Final search: (8 and 9)

Filtro per Revisioni sistematiche

meta-analysis/ or systematic review/ or meta-analysis as topic/ or "meta analysis (topic)"/ or "systematic review (topic)"/ or exp technology assessment, biomedical/

((systematic* adj3 (review* or overview*)) or (methodologic* adj3 (review* or overview*))).ti,ab,kf,kw.

((quantitative adj3 (review* or overview* or synthes*)) or (research adj3 (integrati* or overview*))).ti,ab,kf,kw.

((integrative adj3 (review* or overview*)) or (collaborative adj3 (review* or overview*)) or (pool* adj3 analy*)).ti,ab,kf,kw.

(data synthes* or data extraction* or data abstraction*).ti,ab,kf,kw.

(handsearch* or hand search*).ti,ab,kf,kw.

(meta regression* or metaregression*).ti,ab,kf,kw.

(meta-analy* or metaanaly* or systematic review* or biomedical technology assessment*).mp,hw.

(medline or cochrane or pubmed or medlars or embase or cinahl).ti,ab,hw.

(cochrane or (health adj2 technology assessment) or evidence report).jw.

(comparative adj3 (efficacy or effectiveness)).ti,ab,kf,kw.

(outcomes research or relative effectiveness).ti,ab,kf,kw.

((indirect or indirect treatment or mixed-treatment) adj comparison*).ti,ab,kf,kw.

Combined search: (11 or 12 or 13 or 14 or 15 or 16 or 17 or 18 or 19 or 20 or 21 or 22 or 23 or 24)

Final search: (10 and 25)

((clinical adj3 pathways) or (practice adj3 parameter) or (practice adj3 parameters)).ti,ab,kw. or algorithms/ or care pathway.ti,ab,kw. or care pathways.ti,ab,kw. or clinical protocols/ or Consensus/ or Consensus Development Conference.pt. or Consensus Development Conference, NIH.pt. or Consensus Development Conferences as Topic/ or Consensus Development Conferences, NIH as Topic/ or critical pathway/ or guidance.ti,ab. or guideline*.ti. or guidelines as topic/ or practice guidelines as topic/ or Health Planning Guidelines/ or practice guideline/

Final search: (10 and 27)

Final combined search: (26 or 28)

Filtro per RCT

randomized controlled trial.pt.

controlled clinical trial.pt.

random*.ab.

placebo.ab.

clinical trials as topic.sh.

random allocation.sh.

trial.ti.

Combined search: (10 or 11 or 12 or 13 or 14 or 15 or 16)

Animals exclusion: exp animals/ not humans.sh.

Final search: (39 not 38)

Database: Embase

Data alla ricerca: 1974 to 2020 June 08

Search Query:

Thyroid Nodule/

Nodular goiter/

(thyroi* adj6 (nod* or incidentalom* or goiter)).tw,ot.

((goiter* or goitre*) adj6 (nodul* or multinodul* or multi nodul* or nontoxic or non toxic)).tw.

((thyroid adj3 (neoplasm or tumor)) and benign).tw.

Thyroidectomy/

thyroidectom*.tw.

Combined search: (1 or 2 or 3 or 4 or 5)

Combined search: (6 or 7)

Final search: (8 and 9)

Filtro per revisioni sistematiche

"systematic review"/ or meta analysis/

"meta analysis (topic)"/

"systematic review (topic)"/

biomedical technology assessment/

((systematic* adj3 (review* or overview*)) or (methodologic* adj3 (review* or overview*))).ti,ab.

((quantitative adj3 (review* or overview* or synthes*)) or (research adj3 (integrati* or overview*))).ti,ab.

((integrative adj3 (review* or overview*)) or (collaborative adj3 (review* or overview*)) or (pool* adj3 analy*)).ti,ab.

(data synthes* or data extraction* or data abstraction*).ti,ab.

(handsearch* or hand search*).ti,ab.

(mantel haenszel or peto or der simonian or dersimonian or fixed effect* or latin square*).ti,ab.

(met analy* or metanaly* or technology assessment* or HTA or HTAs or technology overview* or technology appraisal*).ti,ab.

(meta regression* or metaregression*).ti,ab.

(meta-analy* or metaanaly* or systematic review* or biomedical technology assessment*).mp,hw.

(medline or cochrane or pubmed or medlars or embase or cinahl).ti,ab.

(cochrane or (health adj2 technology assessment) or evidence report).jw.

(comparative adj3 (efficacy or effectiveness)).ti,ab.

(outcomes research or relative effectiveness).ti,ab.

((indirect or indirect treatment or mixed-treatment) adj comparison*).ti,ab.

Combined search: (11 or 12 or 13 or 14 or 15 or 16 or 17 or 18 or 19 or 20 or 21 or 22 or 23 or 24 or 25 or 26 or 27 or 28)

Final search: (10 and 29)

exp clinical pathway/

exp clinical protocol/

exp consensus/

exp consensus development conference/

exp consensus development conferences as topic/

critical pathways/

guidelines as topic/

exp practice guideline/

practice guidelines as topic/

health planning guidelines/

(position statement* or policy statement* or practice parameter* or best practice*).ti.

(standards or guideline or guidelines).ti,kw.

((practice or treatment* or clinical) adj guideline*).ab.

consensus*.ti,kw.

((critical or clinical or practice) adj2 (path or paths or pathway or pathways or protocol*)).ti,ab,kw.

recommendat*.ti.

(care adj2 (standard or path or paths or pathway or pathways or map or maps or plan or plans)).ti,ab,kw.

Combined search: (31 or 32 or 33 or 34 or 35 or 36 or 37 or 38 or 39 or 40 or 41 or 42 or 43 or 44 or 45 or 46 or 47)

Final search: (10 and 48)

Final combined search: (30 or 49)

Filtro per RCT

Clinical-Trial/ or Randomized-Controlled-Trial/ or Randomization/ or Single-Blind-Procedure/ or Double-Blind-Procedure/ or Crossover-Procedure/ or Prospective-Study/ or Placebo/

(((clinical or control or controlled) adj (study or trial)) or ((single or double or triple) adj (blind$3 or mask$3)) or (random$ adj (assign$ or allocat$ or group or grouped or patients or study or trial or distribut$)) or (crossover adj (design or study or trial)) or placebo or placebos).ti,ab.

Combined search: (52 or 53)

Final search: (10 and 54)

Database: Web of Science

Search Query:

TS= clinical trial* OR TS=research design OR TS=comparative stud* OR TS=evaluation stud* OR TS=controlled trial* OR TS=follow-up stud* OR TS=prospective stud* OR TS=random* OR TS=placebo* OR TS=(single blind*) OR TS=(double blind*)

TS=(thyroi* NEAR/6 (nod* or incidentalom* or goiter))

TS=((goiter* or goitre*) NEAR/6 (nodul* or multinodul* or nontoxic or "non toxic"))

Combined search: (#2 OR #3)

TI=thyroidectom*

Final search: (#1 AND #4 AND #5)

Database: CINAHL (EBSCO)

Data della ricerca: 1974 to 2020 June 16

Filtro per revisioni sistematiche

Search Query:

( (MH "Random Assignment") or (MH "Random Sample+") or (MH "Crossover Design") or (MH "Clinical Trials+") or (MH "Comparative Studies") or (MH "Control (Research)+") or (MH "Control Group") or (MH "Factorial Design") or (MH "Quasi-Experimental Studies+") or (MH "Placebos") or (MH "Meta Analysis") or (MH "Sample Size") or (MH "Research, Nursing") or (MH "Research Question") or (MH "Research Methodology+") or (MH "Evaluation Research+") or (MH "Concurrent Prospective Studies") or (MH "Prospective Studies") or (MH "Nursing Practice, Research-Based") or (MH "Solomon Four-Group Design") or (MH "One-Shot Case Study") or (MH "Pretest-Posttest Design+") or (MH "Static Group Comparison") or (MH "Study Design") or (MH "Clinical Research+")) or (clinical nursing research or random* or cross?over or placebo* or control* or factorial or sham* or meta?analy* or systematic review* or blind* or mask* or trial*)

(MH "Thyroid Nodule")

TI ( (thyroi* N6 (nod* or incidentalom* or goiter))) OR AB ( (thyroi* N6 (nod* or incidentalom* or goiter)))

TI ( ((goiter* or goitre*) N6 (nodul* or multinodul* or multi nodul* or nontoxic or non toxic))) OR AB ((goiter* or goitre*) N6 (nodul* or multinodul* or multi nodul* or nontoxic or non toxic))

Combined search: (S2 OR S3 OR S4)

(MM "Thyroidectomy")

TI Thyroidectom* OR AB Thyroidectom*

Combined search: (S6 OR S7)

Final search: (S5 AND S8)

Final combined search: (S1 AND S5 AND S8)

Database: Web of Science

Data della ricerca: 1997 to October 13, 2020

Indexes=SCI-EXPANDED, SSCI, A&HCI, CPCI-S, CPCI-SSH, ESCI Timespan=All years

Search Query:

Combined search: (#9 AND #3)

(#8 OR #7 OR #6 OR #5 OR #4)

TS=(lobectomy NEAR/2 (thyroi* or goiter* or goitre*))

TS="Thyroid Lobectomy"

TS=Isthmectomy

TS=hemithyroidectomy

TS=(partial NEAR/2 thyroidectom*)

Combined search: (#2 OR #1)

TS=((goiter* or goitre*) NEAR/6 (nodul* or multinodul* or nontoxic or "non toxic"))

TS=(thyroi* NEAR/6 (nod* or incidentalom* or goiter))

**Supplementary Material 2**

| **Table 1**  **Rates for exams, consultations, interventions, and follow-up procedures (22)** | |
| --- | --- |
| **Service** | **Unit Cost (€)** |
| Specialist consultation | 20.66 |
| Complete blood count (CBC) | 3.17 |
| Fibrinogen | 2.67 |
| Creatinine | 1.13 |
| Prothrombin time (PT) | 2.85 |
| Partial thromboplastin time (PTT) | 2.85 |
| Thyrotropin (TSH) | 5.46 |
| Free thyroxine (FT4) | 6.36 |
| Total calcium | 1.13 |
| Electrocardiogram | 11.62 |
| Neck ultrasound | 28.41 |
| Chest X-ray | 15.49 |
| Thyroid fine-needle aspiration | 86.10 |
| Thyroid cytology | 33.78 |
| Histological examination | 27.17 |
| Laryngoscopy | 27.11 |
| Ordinary hospital stay per day | 674.00 |

| **Table 2**  **Cost of Acquisition for Pharmaceuticals (24)** | |
| --- | --- |
| **Drugs** | **Cost per mg (€)** |
| Betamethasone | 0.1917 |
| Calcium gluconate | 0.0018 |
| Cefazolin | 0.0016 |
| Ceftriaxone | 0.0045 |
| Cortisone | 0.0184 |
| Fentanyl | 3.0400 |
| Hydrocortisone | 2.3187 |
| Lidocaine | 0.0016 |
| Midazolam | 0.1336 |
| Paracetamol | 0.0004 |
| Propofol | 0.0148 |
| Remifentanil | 3.0400 |
| Rocuronium | 0.0661 |
| Ropivacaine | 0.4511 |
| Sodium chloride | 0.0002 |

| **Table 3**  **Cost per Minute for Health Professionals and Operating Room Use (25,26)** | |
| --- | --- |
| **Health Professional / Resource** | **Cost per minute (€)** |
| Physician | 0.58 |
| Nurse | 0.27 |
| Health assistant | 0.23 |
| Speech therapist | 0.25 |
| Operating room | 20.43 |

| **Table 4**  **Unit Cost of Materials** | |
| --- | --- |
| **Material** | **Cost per unit (€)** |
| Needle | 0.06 |
| Venous catheter | 35.00 |
| Bi-Clamp forceps | 25.03 |
| Electrosurgical knife | 14.34 |
| Scalpel | 15.38 |
| Surgical strips | 0.18 |
| Needle electrode | 40.00 |
| Gauze | 0.10 |
| Surgical swab | 2.20 |
| Dressing 8x15 | 0.54 |
| Suture thread | 6.00 |
| Surgical drape | 2.16 |
| Sterile material pack | 24.52 |
| Hemovac drain | 6.50 |
| Vicryl suture | 6.00 |
| Monocryl suture | 7.25 |
| Ice pack | 6.21 |
| Jackson-Pratt drain | 3.07 |
| Ultrasound machine | 56.00 |
| Radiofrequency needle electrode | 1,240.00 |
| ECG monitoring electrodes | 0.04 |
| Optical fiber | 330.00 |

| **Table 5**  **Hourly Wage by Job Class and Distribution of Caregivers by Job Class** | | | |
| --- | --- | --- | --- |
| **Job Class** | **Annual Income (€)** | **Hourly Income (€) (Assuming 40-hour workweek)** | **% of Caregivers in Each Job Class** |
| Managers | 101,096.00 | 48.60 | 1.3% |
| Mid managers | 54,136.00 | 26.03 | 4.4% |
| Employees | 30,770.00 | 14.79 | 36.0% |
| Workers/Apprentices | 24,780.00 | 11.91 | 58.3% |
